# Pharmacokinetic–pharmacodynamic modelling of the hypoglycaemic effect of pulsatile administration of human insulin in rats

**DOI:** 10.1038/s41598-020-76007-3

**Published:** 2020-11-02

**Authors:** Makoto Miyazaki, Mariko Hayata, Noriaki Samukawa, Kazunori Iwanaga, Junya Nagai

**Affiliations:** Department of Pharmaceutics, Education and Research Center for Pharmaceutical Sciences, Osaka University of Pharmaceutical Sciences, 4-20-1 Nasahara, Takatsuki, Osaka 569-1094 Japan

**Keywords:** Drug therapy, Pharmaceutics, Pharmacokinetics, Pharmacodynamics

## Abstract

The relationship between the plasma insulin (INS) concentration–time course and plasma glucose concentration–time course during and after pulsatile INS administration to rats was characterized using a pharmacokinetic–pharmacodynamic (PK–PD) model. A total INS dose of 0.5 IU/kg was intravenously injected in 2 to 20 pulses over a 2-h period. Compared with the single bolus administration, the area under the effect-time curve (AUE) increased depending on the number of pulses, and the AUEs for more than four pulses plateaued at a significantly larger value, which was similar to that after the infusion of a total of 0.5 IU/kg of INS over 2 h. No increase in plasma INS concentration occurred after pulsatile administration. Two indirect response models primarily reflecting the receptor-binding process (IR model) or glucose transporter 4 (GLUT4) translocation (GT model) were applied to describe the PK–PD relationship after single intravenous bolus administration of INS. These models could not explain the observed data after pulsatile administration. However, the IR-GT model, which was a combination of the IR and GT models, successfully explained the effects of pulsatile administration and intravenous infusion. These results indicate that the receptor-binding process and GLUT4 translocation are responsible for the change in AUE after pulsatile administration.

## Introduction

Glycaemic homeostasis is tightly regulated by various mechanisms, and insulin (INS) plays a central role in maintaining glycaemic homeostasis. In patients with type 1 diabetes, β cells in the pancreatic islets of Langerhans undergo apoptosis or necrosis, and INS secretion is extremely reduced. In the clinical treatment of patients with type 1 diabetes, INS is frequently administered by several subcutaneous injections or continuous subcutaneous infusion^1–3^. This is termed intensive INS therapy. The INS injection dose is adjusted by the patient according to the doctor’s instructions to achieve the best possible glycaemic control. However, the higher incidence of hypoglycaemia and the cost of treatment required to achieve strict control may be problematic.

In vivo, peptide hormones, such as INS, are stored in intracellular granules and rapidly secreted as needed. β cells in the pancreas secrete INS as discrete pulses every few minutes. In the fasted state, a pulse of INS is secreted every 5 to 15 min in humans^4–6^ and every 4 to 12 min in rats^7,8^. Secreted INS enters the general circulation from the portal vein via the liver and is transported to tissues throughout the body to exert its effects. However, the method of INS administration in intensive INS therapy significantly differs from endogenous secretion. As a result, the dose may be excessive and result in hypoglycaemia. Pharmacokinetic–pharmacodynamic (PK–PD) models have been reported in efforts to better understand exogenous INS dosing and optimise treatments^9,10^. However, these models focus on single or subcutaneously administered doses and have not been used to investigate pulsed dosing.

In this study, we administered INS in pulses by repeated intravenous bolus injections. In addition, the quantitative characterization of the hypoglycaemic effect of pulsatile administration was performed. Generally, one PK–PD relationship is established between the PK of a drug and its pharmacological effect, and this relationship is independent of the administration method. In this study, we investigated the PK–PD relationship after a single bolus administration, assuming that the relationship was not changed by pulsatile administration. Therefore, we aimed to construct a PK–PD model after a single intravenous bolus administration in rats. Using this model, we predicted the plasma INS concentration and hypoglycaemic effect after pulsatile administrations and compared these with the observed data.

## Results

### PK and pharmacological effects of pulsatile INS administration

**Figure 1:**
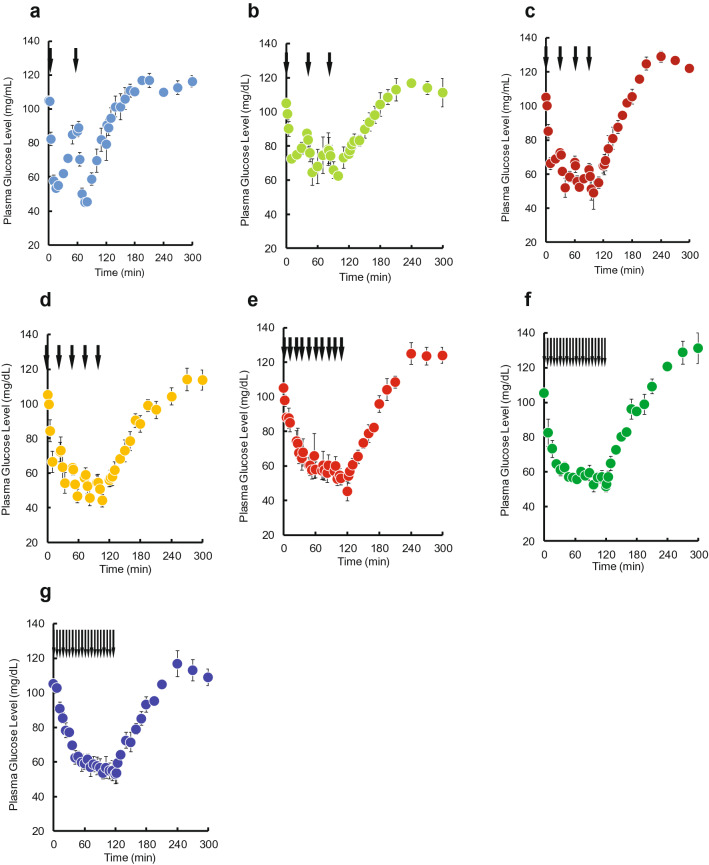
The time courses of plasma glucose concentration during and after intravenous pulsatile administration of INS (0.5 IU/kg) in rats. **(a)** 2 pulses, **(b)** 3 pulses, **(c)** 4 pulses, **(d)** 5 pulses, **(e)** 10 pulses, **(f)** 15 pulses, and **(g)** 20 pulses. The arrows indicate the dosing times. The points represent the mean ± S.E., n = 3–4.

**Figure 2:**
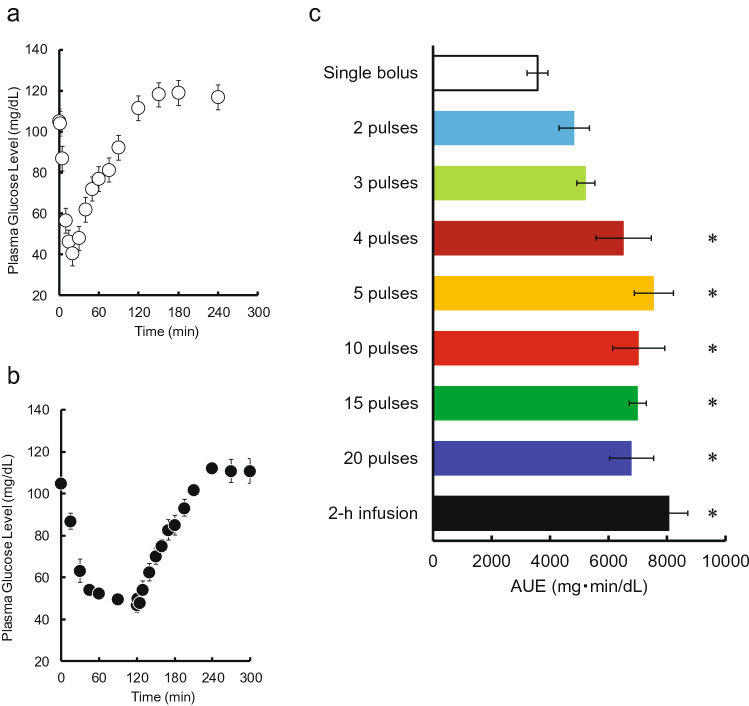
**(a)** The time courses of plasma glucose concentration after a single intravenous bolus administration of INS (0.5 IU/kg) in rats. **(b)** The time courses of plasma glucose concentration during and after intravenous infusion administration of INS (0.5 IU/kg) for 2 h in rats. The points represent the mean ± S.E., n = 3–4. **(c)** The effects of the pulse rate of administration on the AUE after intravenous administration of INS (0.5 IU/kg/2 h) in rats. The data represent the mean ± S.E., n = 3–4. *p < 0.05 compared with single bolus administration by Dunnett’s test.

Time courses of plasma glucose concentration during and after pulsatile administration of INS are shown in Fig. 1. A decrease in plasma glucose concentration was observed when INS was injected. No increases in the basal or maximum concentrations of plasma INS during was observed during the pulsatile administration (see Supplementary Fig. S1 online). Time courses of plasma glucose concentration during and after intravenous bolus injection and 2-h infusion of INS are shown in Fig. 2a,b. A substantial decrease was observed after the bolus injection. The glucose level gradually decreased after the start of infusion and then recovered to its basal level after the end of infusion. Despite the same total dose (0.5 IU/kg) being administered within the 2-h period, the area between the effect-time curve and the basal concentration (AUE) values was significantly higher than following the bolus injection of 0.5 IU/kg, and the magnitude of the difference depended on the pulse rate (Fig. 2c). The increase was saturated when more than four injections were administered, and the maximum value of AUE for pulsatile administration was equal to that for the infusion.

### PK modelling of plasma INS after bolus administration

**Figure 3:**
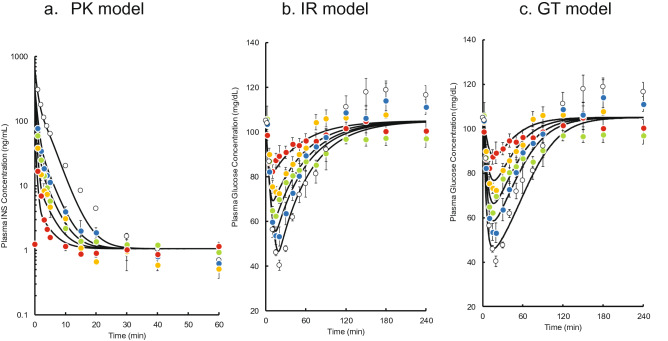
The time courses of plasma INS **(a)** and glucose **(b, c)** concentrations after a single intravenous bolus administration of INS in rats. The doses are 0.05, 0.1, 0.17, 0.25, and 0.5 IU/kg (red, yellow, green, blue, and white, respectively). The points represent the mean ± S.E., n = 3–5. The solid lines are the theoretical curves fitted to the PK model **(a)**, IR model **(b)**, and GT model **(c)**, respectively.

**Figure 4:**
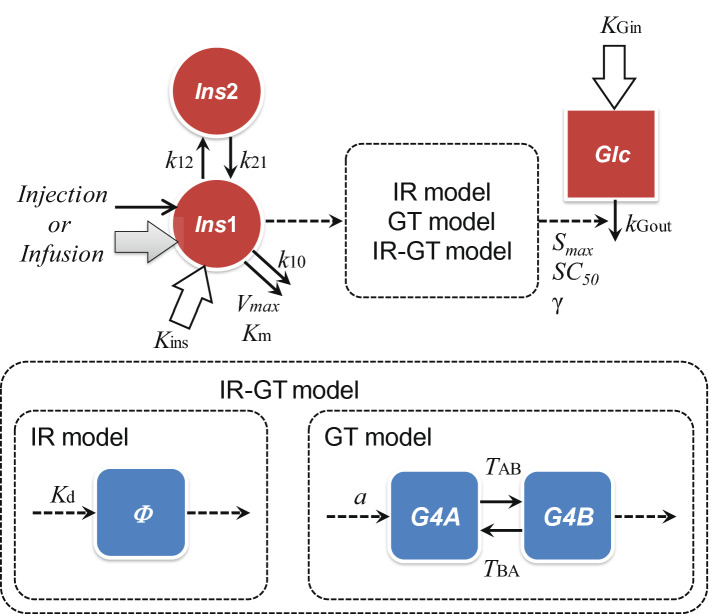
Diagrammatic representation of the PK–PD model for the hypoglycaemic effect. The plasma INS concentration is described by a nonlinear two-compartment open model with Michaelis–Menten type and linear elimination kinetics and zero-order biosynthetic kinetics. *Ins1* and *Ins2* are the amounts of insulin in the central and peripheral compartments (ng/kg), respectively. *k*_*12*_ and *k*_*21*_ are the first-order rate constants (min^-1^) between the central and peripheral compartments, *k*_10_ is the first-order rate constant (min^-1^) for the linear elimination of INS, V_max_ (ng/kg/min) and K_m_ (ng/kg) are Michaelis–Menten kinetic constants, and K_ins_ is the zero-order constant for the endogenous synthesis of INS. An indirect response model was used to analyse the PK-PD relationship. Glc is the plasma glucose concentration (mg/dL), S_max_ is the maximum effect, SC_50_ is the theoretical signal strength producing 50% of S_max_, K_Gin_ is the zero-order rate constant for endogenous glucose production, and *k*_Gout_ is the first-order rate constant (min^-1^) for glucose elimination. IR and GT models and their combination, the IR-GT model, were used. K_d_ is the dissociation constant for the receptor and Φ is the ratio of receptor occupancy by INS. G4A is the hypothetical amount of inactive GLUT4 in the intercellular pool, G4B is the hypothetical amount of active GLUT4 on the cell surface, and α is the transit coefficient between the plasma INS concentration and stimulation for translocation. *T*_AB_ is the time is takes for G4A to translocate to the cell surface and *T*_BA_ is the time over which cells take up glucose from plasma.

Time courses of plasma INS concentration after single bolus administration of INS are shown as plotted points in Fig. 3a. A two-exponential decline in the INS level occurred after every dose (0.05–0.5 IU/kg) of INS. Because the dose-normalized area between the plasma INS concentration–time curve and the basal concentration (AUC) increased significantly with the dose of INS (0.0117 ± 0.000978 min·kg/mL at 0.05 IU/kg vs 0.0541 ± 0.0109 min·kg/mL at 0.5 IU/kg, p = 0.0057, Student’s t-test), the plasma concentrations of INS were fitted to a two-compartment model with Michaelis–Menten type and first-order elimination kinetics (Fig. 4). The lines shown in Fig. 3a represent the results of the least-squares regression fitting of the observed data to the model using Eqs. (1–4), and the estimated PK parameters are listed in Supplementary Table S1 online.

### PK–PD modelling of the bolus administration of INS using IR and GT models

Because plasma glucose concentrations indicated the presence of a delay in the hypoglycaemic effect of increasing plasma concentration (Fig. 3a–c), an indirect response model was applied to the PK–PD model analysis. The PD model combined with a PK model (the IR model or GT model) was used to represent the insulin receptor binding process or glucose transporter 4 (GLUT4) translocation process, respectively (Fig. 4). The simultaneous fitting of the plasma glucose data after single bolus administration (0.05–0.5 IU/kg) to the IR model (Fig. 3b) or GT model (Fig. 3c) successfully described the observed data. The estimated PD parameters are listed in Supplementary Table S1 online.

### Simulation of the pharmacological effect after pulsatile administration and infusion

**Figure 5:**
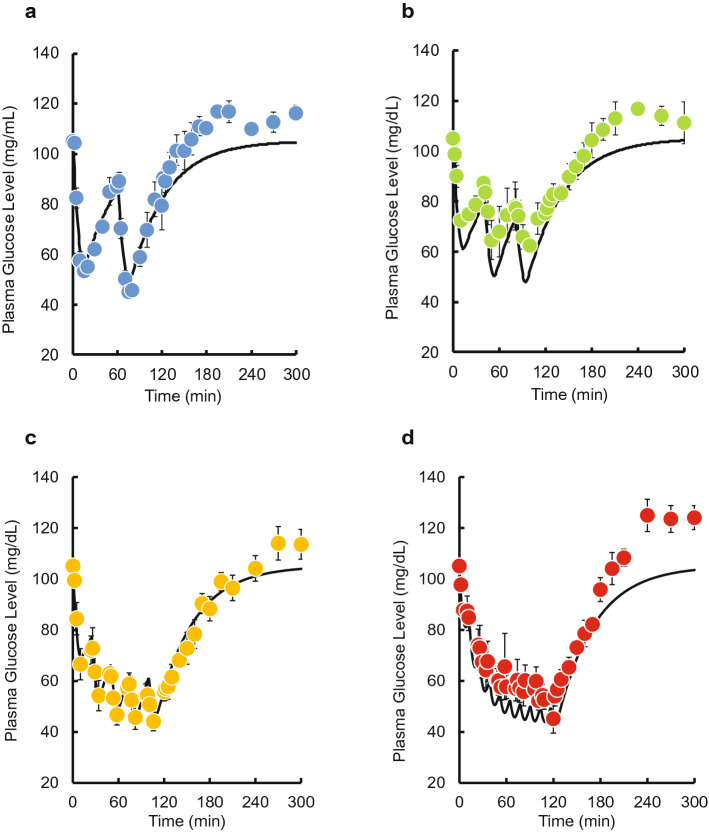
Comparison of the IR model-simulated plasma glucose concentration–time courses and the observed data during and after intravenous pulsatile administration of INS (0.5 IU/kg) in rats. **(a)** 2 pulses, **(b)** 3 pulses, **(c)** 5 pulses, **(d)** 10 pulses. The solid lines are the predicted curves from the IR model. The points represent the observed data and the mean ± S.E., n = 3–4.

**Figure 6:**
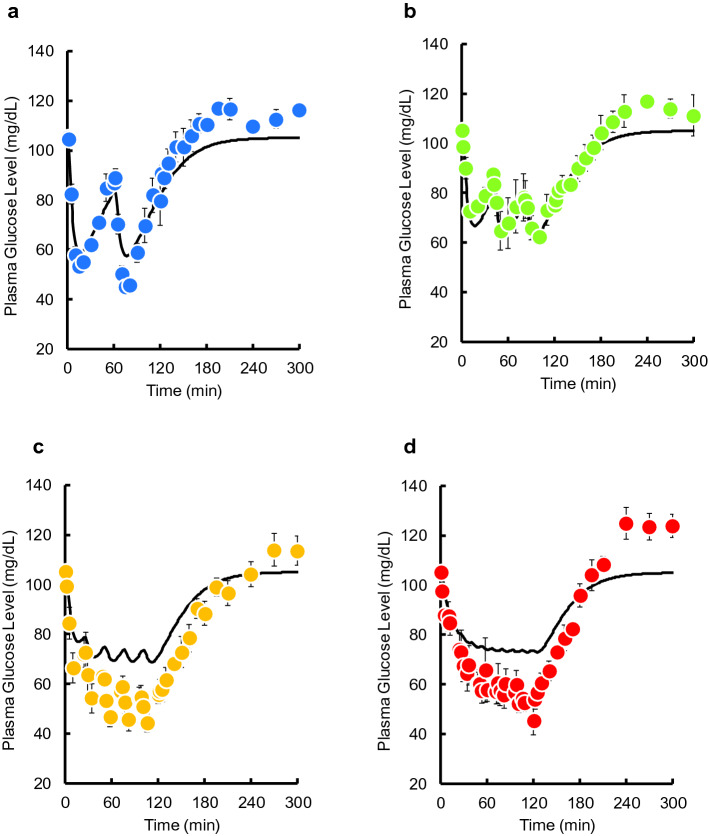
Comparison of the GT model-simulated plasma glucose concentration–time courses and the observed data during and after intravenous pulsatile administration of INS (0.5 IU/kg) in rats. **(a)** 2 pulses, **(b)** 3 pulses, **(c)** 5 pulses, **(d)** 10 pulses. The solid lines are the predicted curves from the GT model. The points represent the observed data and the mean ± S.E., n = 3–4.

**Figure 7:**
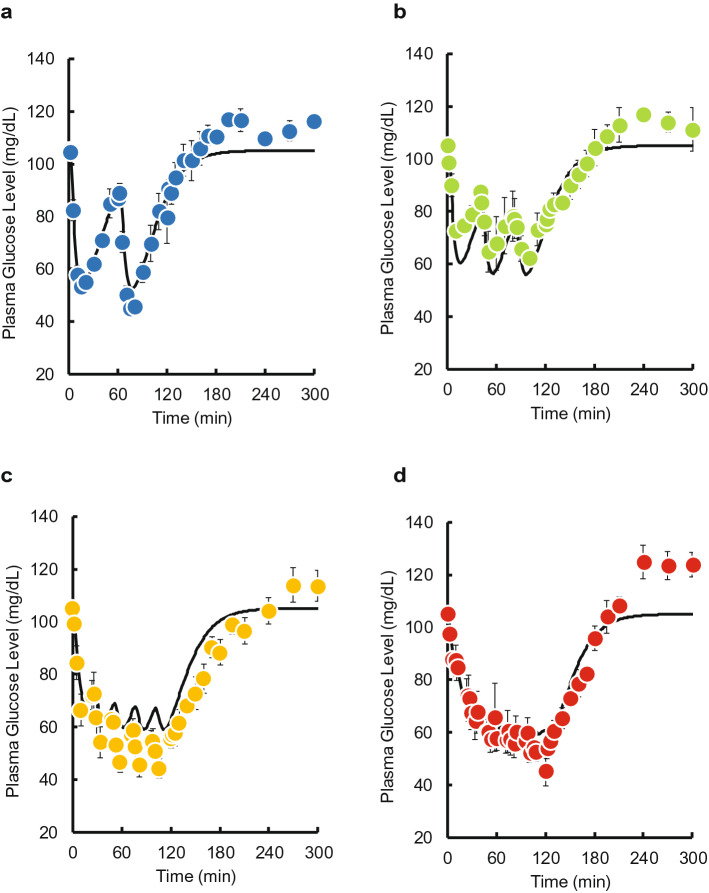
Comparison of the IR-GT model-simulated plasma glucose concentration–time courses and the observed data during and after intravenous pulsatile administration of INS (0.5 IU/kg) in rats. **(a)** 2 pulses, **(b)** 3 pulses, **(c)** 5 pulses, **(d)** 10 pulses. The solid lines are the predicted curves from the IR-GT model. The points represent the observed data and the mean ± S.E., n = 3–4.

**Figure 8:**
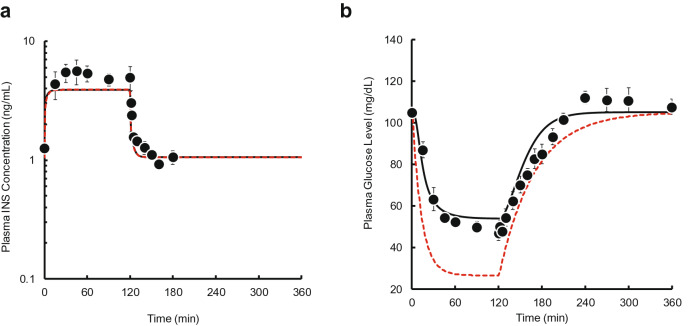
Comparison of the IR-GT model-simulated plasma INS **(a)** and glucose **(b)** concentration–time courses and the observed data during and after intravenous infusion administration of INS (0.5 IU/kg/2 h) in rats. The solid and dashed lines are the predicted curves from the IR-GT model and a typical indirect response model without the IR and GT models, respectively. The points represent the observed data and the mean ± S.E., n = 3–4.

Time courses of plasma INS and glucose concentrations after two, three, five, and ten pulses were simulated using the PK and PD parameter estimates. The predicted plasma INS concentrations closely mirrored the observed data (see Supplementary Fig. S1 online). However, the predicted plasma glucose concentrations using the IR model slightly overestimated the effect of INS on plasma glucose level compared with the observed data (Fig. 5), whereas the GT model underestimated the effect of INS (Fig. 6). Therefore, we constructed an IR-GT model to represent both GLUT4 translocation and the receptor-binding process (Fig. 4). The fitting of the plasma glucose data after single bolus administration (0.05–0.5 IU/kg) to the IR-GT model resulted in a good fit with the observed data; see Supplementary Fig. S2 online. In addition, the Akaike Information Criterion (AIC) and sum of squares (SS) values for all of the models at the time of fitting are given in Supplementary Table S2 online. There was no significant difference between the four models, indicating that they all explain the observations to the same extent. The estimated PD parameters are listed in Supplementary Table S3 online. Using these parameter estimates, time courses of plasma glucose concentrations during and after the pulsatile administration of INS were simulated to compare with the observations (Fig. 7). The model successfully represented data for 2–10 exogenous INS pulses. In the IR-GT model, the mean errors (ME) of the predicted values for both the bolus and 10-INS pulse regimen included 0 in the 95% confidence intervals (see Supplementary Table S4 online). Both the mean absolute error (MAE) and root mean square error (RMSE) values were lower for the IR-GT model than for the other models. To assess the validity of the IR-GT model, we simulated the 2-h infusion administration of INS at a dose rate of 0.25 IU/kg/h (Fig. 8, solid lines) and compared the results with the observed data (Fig. 8, points). Both the predicted time courses of plasma INS and glucose concentrations successfully described the observed data. However, predictions made using the typical indirect response model, rather than with the IR or GT models (see Supplementary Fig. S3 and Table S5 online), clearly overestimated the plasma glucose concentration (Fig. 8b).

## Discussion

There have been numerous attempts to apply modelling approaches to the response of blood glucose to INS dosing to optimise therapeutic interventions for diabetic patients. For example, Campos-Náñez et al. recently used an in silico model to determine changes in blood glucose resulting from variations in the basal rate or the magnitude of bolus doses^11^. Other examples include a machine learning approach used by Seo et al. to predict hypoglycaemia to improve the understanding of INS infusion and continuous glucose monitoring^12^. Effective PK–PD models have been reported based on inhaled and subcutaneously administered or single INS doses in healthy and diabetic volunteers^9,10,13^; however, the models have not been used to study intravenous pulsed administration.

This work, which extended the previous PK–PD models, determined two characteristics of the hypoglycaemic effect of the investigated pulsatile administration of INS. First, the AUE increases depending on the pulse rate; however, the increase saturates after four or more pulses. Second, the IR-GT model can explain the change in the plasma glucose concentration–time course during both the pulsatile administration and infusion administration using the PK–PD relationship after the bolus administration.

No accumulation in plasma INS concentration during pulsatile administration was observed. Therefore, the pulse rate-dependent AUE was due to PD factors, not PK factors. It has been reported that AUE is not always proportional to the dose^14^. In addition, it has been suggested that even if the dose is the same, AUE is higher at a slower dosing rate. In the pulsatile administration in this study, the dose per unit time was equal because the dose per 2 h was equal (the dose per pulse decreased depending on the number of pulses, whereas the AUE increased). However, the AUE was not necessarily equal. Therefore, changes in the apparent dose rate or dose per pulse cannot explain the differences in AUE. The typical indirect response model overestimated the hypoglycaemic effect after infusion. These findings suggest that there is a PK–PD relationship that cannot be explained solely by the observed PK–PD relationship after bolus administration.

In the post-prandial state, the uptake of glucose from the blood in vivo mainly occurs in skeletal muscle, which expresses INS receptors. In the IR model, 2.06 nmol/L was used as the dissociation constant (*K*d) in rat soleus muscle cells^15^. Although there are various kinds of skeletal muscle in the body, it would not be useful to individually model each receptor in all skeletal muscles, because this complicates the model. In addition, even though the binding constant for soleus muscle cells is lower than that for other skeletal muscles, the number of binding sites here is reported to be the highest of the skeletal muscles investigated^15^. In the model used in this study, the relationship between INS concentration and receptor binding was represented by these characteristics of soleus myocytes. However, the PK–PD relationship based on the IR model alone could not explain the changes in plasma glucose concentration–time courses during all the types of pulsatile administration.

INS bound to the receptor stimulates GLUT4 internalized in the cell, which translocates to the cell surface where it takes up glucose to lower the plasma glucose level^16^. The pulse intervals investigated in this study were 1 h for 2 pulses, 40 min for 3 pulses, 30 min for 4 pulses, 24 min for 5 pulses, 12 min for 10 pulses, 8 min for 15 pulses, and 6 min for 20 pulses. Infusion can be considered the minimum pulse interval. In the GT model, we assumed that the time taken to internalize GLUT4 from the cell surface (T_BA_) was 20 min^17,18^, which is long compared with the assumption that GLUT4 localizes to the cell surface in 3 min (T_AB_)^17,18^. The values for T_BA_ and T_AB_ were selected after considering the review by Foley et al. that summarizes the numerous values that have been reported. It should be noted that we have chosen to base our model on the quantitative data available for GLUT4 translocation. Although other potentially rate-determining processes such as glucose phosphorylation may also be included in T_AB_/T_BA_, quantitative data for these processes are currently unavailable. We have therefore chosen to describe our model in terms of GLUT4 translocation, although the GT model has the potential to include glucose phosphorylation. Because the T_BA_ value coincides with the pulse interval time of four to five pulses, the saturation of AUE within four pulses may be related to this T_BA_ value. The GLUT4 translocation process might contribute to the PK–PD relationship during pulsatile administration and the saturation of AUE, although the GT model alone could not explain the differences in the plasma glucose concentration–time courses during the types of pulsatile administration.

The IR-GT model was therefore used to try to improve the prediction of plasma glucose concentration–time courses after pulsatile administration, and was found to be effective, suggesting that both the IR and GT models were important factors for the hypoglycaemic effect of INS and were closely related to the PK–PD relationship. In this model, the maximum values for receptor occupancy were about 88%, 82%, 76%, 63%, 40%, and 16% for one, two, three, five, and ten pulses, and infusion administration, respectively, which reflects the decrease in the dose contained in an individual pulse. AUE increased with 1–5 pulses despite a decrease in receptor occupancy, while the AUE was constant at five and ten pulses and for infusion, despite the decrease in receptor occupancy. The change in plasma INS concentration is rapid, and the binding of INS to the receptor occurs instantaneously^19^. In contrast, the time required for the maximal hypoglycaemic effect after bolus administration is about 20 min (Fig. 3). It has also been shown that the translocation of GLUT4 is the rate-limiting step in the mechanism contributing to the hypoglycaemic effect of INS^20–22^. Therefore, the enhancement of the hypoglycaemic effect based on the time delay required for the plasma glucose uptake process may be the cause of the change in AUE.

A comparable situation has been reported for leukopenia—which is a side effect of anticancer drugs that correlates with the AUC of the drugs. To represent this relationship, there are several models of the PK–PD relationship that incorporate AUC directly rather than the plasma drug concentration–time course^23,24^. However, Krzyzanski and Jusko successfully demonstrated leukopenia by modelling the site of action of anticancer drugs and the cell lifespan^25^. The model only represented the relationship between the plasma drug concentration and white blood cell count and did not describe the effect of the AUC on the white blood cell count. However, the model was able to represent an AUC-dependent decrease in white blood cell count as a result of the cell lifespan modelling. The IR-GT model also schematically illustrates only the INS receptor response and GLUT4 translocation and intentionally does not incorporate the pulse rate into the model. However, as a result, pulse-dependent increases and the saturation of AUE could be explained. The PK–PD relationship after a single bolus administration based on a typical mode could not predict the change in plasma glucose concentration after pulsatile administration. This suggests that aspects of both of the IR and GT models are closely related to the PK–PD of INS.

Our findings imply that pen-type self-injection devices for INS administration may be able to achieve the same effects as continuous dosing if the dosing interval is controlled. Conversely, our findings suggest that inadvertent repeated doses can lead to unexpected hypoglycaemia. The PK–PD of INS was found to be clearly different for bolus doses compared with continuous administration. In addition, depending on the dose rate, the PK–PD relationship may be an important consideration for drug development. Therefore, the simple structure of the demonstrated model, which can describe the PK–PD of INS with a single model and a single set of parameters, is a useful starting point for the management of therapeutic interventions.

The biochemistry of INS has been widely studied and reported and many biological and biochemical findings have been published as qualitative results in vitro; however, less is known regarding how these findings translate to the in vivo situation. This study showed that the plasma glucose-lowering effect of pulsatile INS administration is closely related to processes such as the insulin receptor response and GLUT4 translocation. The model also illustrates that these processes are quantitatively continuous. It should be noted that the insulin receptor response and GLUT4 translocation in the model do not actually refer to specific tissues or organs, receptors, transporters, or enzymes, as it is well known that tissues other than muscle contribute to the reduction of glucose by insulin. However, the purpose of this study was to establish a simple model of the action of insulin in a pulse-dependent manner with the minimum necessary factors, and insulin receptor response and GLUT4 translocation provide a good indication of which process is necessary to fit the observations.

In summary, the hypoglycaemic effect of INS increases depending on the pulse rate, and it was shown that pulsatile administration of boluses at intervals of 20 to 30 min could lead to an effective hypoglycaemic effect, similar to that of infusion. This mechanism is suggested to be related to the INS receptor-binding process and GLUT4 translocation. Although there is room for a more detailed study, pulsatile administration based on the pharmacological mechanism of INS can achieve an effect comparable to infusion, even when a device capable of bolus administration is used. The findings of this study may therefore provide important information for dose preparation when designing dosing devices. In addition, the main action of recently developed diabetes drugs such as dipeptidyl peptidase 4 inhibitor and glucagon-like peptide-1 receptor agonist is to induce the secretion of endogenous insulin to lower the plasma glucose level; this study may provide useful insight for determining strategies and policies in the development of drugs with these novel pharmacological mechanisms.

## Materials and methods

### Chemicals

Human recombinant INS (Humulin R) was purchased from Eli Lilly Japan K.K. All other reagents and solvents were commercially available reagents.

### Animal experiments

Animal experiments were performed following the procedures as described earlier^26^, with slight adaptations. In short, male Wistar rats (Japan SLC, Shizuoka, Japan) weighing 270–320 g were used in this study. Rats were housed in controlled environmental facilities (temperature: 24 ± 1 °C, humidity: 55% ± 10%) under a 12:12-h light–dark cycle (06:00 h/18:00 h) for more than 1 week and allowed free access to a standard diet and tap water. One day before the experiment, rats were anaesthetized with ethyl ether and implanted surgically with a phycon tube connected to a PE50 catheter in the jugular vein for drug administration and another phycon-PE50 catheter in the femoral vein for blood sampling. Both catheters were externalized through the back in the neck region and secured. Unless otherwise specified, all animal experiments were conducted without restraint or anaesthesia and in a fasted state. On the day of the experiment, each rat was housed in an individual cage and left alone for at least 1 h. INS was dissolved in a 0.1% bovine serum albumin solution. Pulsatile administration of INS was performed by repeated intravenous bolus injections at constant intervals of 6–60 min within 2 h (total dose: 0.5 IU/kg). As controls, a single bolus injection or 2-h infusion were also conducted at doses of 0.5 IU/kg and 0.25 IU/kg/h, respectively. INS (0.025–0.5 IU/kg) was intravenously injected to estimate PK and PD parameters. Blood samples (≤ 0.1 mL) were withdrawn from the femoral vein before and at designated times after dosing. The blood samples were transferred to heparinized tubes, then centrifuged (12,000 × *g*, 5 min). The isolated plasma was stored frozen at − 20 °C until analysis. Immunoreactive INS concentrations in plasma were determined using an enzyme-linked immunosorbent assay (ELISA) kit (Shibayagi, Gunma, Japan). The mutarotase glucose oxidase method kit (Wako Pure Chemical Industries, Osaka, Japan) was used to measure plasma glucose concentrations. These animal experiments were performed in accordance with the relevant guidelines and regulations and approved by the Animal Experimentation Committee of Osaka University of Pharmaceutical Sciences (Approval numbers 10010 and 11008).

### Theoretical

The plasma concentrations of INS were described by a nonlinear two-compartment open model with Michaelis–Menten type and linear elimination kinetics and zero-order biosynthetic kinetics as follows:

1 $$\frac{dIns1}{dt}={K}_{ins}+{k}_{21}\bullet Ins2-\left({k}_{12}+{k}_{10}+\frac{{V}_{max}}{{K}_{m}+Ins1}\right)\bullet Ins1,$$

2 $$\frac{dIns2}{dt}={k}_{12}\bullet Ins1-{k}_{21}\bullet Ins2,$$

3 $${C}_{Ins}=\frac{Ins1}{Vc}.$$

*Ins*1 and *Ins*2 are the amounts of INS in the central (plasma) and peripheral compartments (ng/kg), respectively, *k*_12_ and *k*_21_ are the first-order rate constants (min^-1^) between the central and peripheral compartments, *k*_10_ is the first-order rate constant (min^-1^) for the linear elimination of INS, *V*_max_ (ng/kg/min) and *K*_m_ (ng/kg) are Michaelis–Menten kinetic constants, *C*_ins_ is the plasma concentration of INS (ng/mL), *V*c is the distribution volume of the central compartment (mL/kg), and *K*_ins_ is the zero-order constant for the endogenous synthesis of INS. At basal steady state before INS administration, *K*_ins_ is characterized by Eq. (4), because the influx of INS to the central compartment is equal to the efflux from the compartment.

4 $${K}_{ins}=\frac{{C}_{00}\bullet Vc\bullet {V}_{max}}{{K}_{m}+{C}_{00}\bullet Vc}+\left({C}_{00}\bullet Vc\bullet {k}_{10}\right).$$

*C*_00_ is the observed mean INS concentration in plasma (1.06 ng/mL). At *t* = 0, *Ins*1 = *C*_00_.*V*c + *D*, where D is the pulse dose of insulin (ng/kg).

An indirect response model was used to analyse the PK–PD relationship. The differential equation for the INS stimulus of the model is as follows:

5 $$\frac{dGlc}{dt}={K}_{Gin}-\left(1+\frac{{S}_{max}\bullet {SIG}^{\gamma }}{{{SC}_{50}}^{\gamma }+{SIG}^{\gamma }}\right){k}_{Gout}\bullet Glc,$$

where *Glc* is the plasma glucose concentration (mg/dL), *SIG* is the theoretical signal strength concerned with the hypoglycaemic effect of INS, *S*_max_ is the maximum effect, *SC*_50_ is the *SIG* producing 50% of *S*_max_, *r* is Hill’s constant, *K*_Gin_ is the zero-order rate constant concerned with endogenous glucose production, and *k*_Gout_ is the first-order rate constant (min^−1^) concerned with glucose elimination. Before INS is administered, the observed baseline level of *Glc* is maintained as *G*_00_ (105.12 mg/dL), which is determined by *K*_Gin_ = *k*_Gout_.*G*_00_. Equation (5) can comprehensively express the mechanism of action of INS that is not assumed in the following model.

To assess the contribution of the receptor-binding process of INS to the hypoglycaemic effect, the INS receptor-based PK–PD model (IR model) was designed as follows:

6 $$\upphi =\frac{{C}_{Ins\bullet 100}}{Kd+{C}_{Ins}}-{\phi }_{00}.$$

*K*d is the dissociation constant for the receptor, and 2.062 nmol/L was used for the rat soleus muscle^10^, *Φ* is the ratio of receptor occupancy by INS, *Φ*_00_ is the basal *Φ* value before INS administration, which was estimated as 8.12%, based on *K*d, *C*_00_, and the molecular weight of INS (ca. 5800). We assumed that *SIG* is equal to the value of *Φ* in this model. To evaluate the contribution of the translocation process to the hypoglycaemic effect, the GLUT4 translocation-based PK–PD model (GT model) was designed as follows:

7 $$\frac{dG4A}{dt}=\alpha \bullet \left({C}_{Ins}-{C}_{00}\right)-\frac{G4A}{{T}_{AB}},$$

8 $$\frac{dG4B}{dt}=\frac{G4A}{{T}_{AB}}-\frac{G4B}{{T}_{BA}}.$$

*G4A* is the hypothetical amount of inactive GLUT4 in the intercellular pool, *G4B* is the hypothetical amount of active GLUT4 on the cell surface, and α is the transit coefficient between the plasma INS concentration and stimulation for translocation. In this model, we assumed that INS immediately stimulates *G4A* in an intracellular pool depending on an increase in the plasma INS concentration, then *G4A* translocates to the cell surface and is activated (*G4B*) after time *T*_AB_. Increased *G4B* causes greater uptake of glucose from the plasma into cells to reduce the plasma glucose concentration throughout time *T*_BA_. After that, *G4B* returns to the intercellular pool; however, the recycled *G4B* has no effect on the amount of *G4A* because of the large amount of inactive GLUT4 in the intercellular pool. We used 3 min and 20 min for time *T*_AB_ and *T*_BA_, respectively, as previously reported^18^. We assumed that *SIG* is equal to the value of *G4B* in this model. The PK–PD model combined with the IR model and the GT model (IR-GT model) was also constructed to evaluate our hypothesis. This model concerns the pharmacological processes of INS, which represents GLUT4 translocation according to an INS receptor response. In this model, Eq. (7) is replaced by Eq. (9):

9 $$\frac{dG4A}{dt}=\phi -\frac{G4A}{{T}_{AB}}.$$

*G4B* was also applied as *SIG* in this model. The parameter values of *S*_max_, *SC*_50_, *r*, and *k*_Gout_ were reanalysed according to Eqs. (1)–(6), (8), and (9). In the simulation of the infusion administration using the IR-GT model, Eq. (1) is replaced by Eq. (10):

10 $$\frac{dIns1}{dt}={K}_{ins}+{k}_{21}\bullet Ins2-\left({k}_{12}+{k}_{10}+\frac{{V}_{max}}{{K}_{m}+Ins1}\right)\bullet Ins1+{K}_{inf},$$

where *K*_inf_ is the infusion rate of INS (IU/min/kg). At *t* = 0, *Ins*1 = *C*_00_.*V*c. To compare with the IR-GT model, a typical PK-PD model based on an indirect response model without IR and GT models (Eqs. (2)–(5) and (10)) was similarly constructed as a control model where *SIG* = *C*_INS_, and we simulated the pharmacological effect after the infusion of 0.5 IU/kg/2 h.

### Modelling, simulation, and data analysis

The concentration–time data were analysed by the nonlinear regression program FKDM^27^. The differential equations were solved by the Runge–Kutta–Gill method. The procedure of data analysis was as follows: (1) plasma INS concentration–time data after the single bolus injection of INS (0.05–0.5 IU/kg) were fitted to a nonlinear two-compartment open model using Eqs. (1)–(4); (2) Using the PK parameter estimates, plasma glucose concentration–time data after the single bolus injection (0.05–0.5 IU/kg) were fitted to the IR model (Eqs. (1)–(6)) and GT model (Eqs. (1)–(4) and (7), (8)), and the PD parameters were determined; (3) plasma INS and glucose concentrations during and after pulsatile administration were predicted by the IR model and the GT model; (4) the glucose-time data (0.05–0.5 IU/kg) after the single bolus injection were also fitted to the IR-GT model (Eqs. (1)–(6), (8), and (9)), and the PD parameters were newly determined; and (5) INS and glucose concentrations in plasma during and after pulsatile administration (2–10 pulses of 0.5 IU/kg) were predicted by the IR-GT model, and the plasma glucose concentration profile during and after intravenous 2-h infusion of INS (0.5 IU/kg/2 h) was simulated using the determined PK and PD parameter estimates of the IR-GT model. Unless otherwise specified, the AUC was calculated to include the last measurement of the concentration–time data by the linear trapezoidal method, and AUCs extending beyond the last data sampling point were estimated by dividing the final concentration–time data by the terminal slope. The AUE between the plasma glucose concentration–time curve and the basal concentration was estimated using the linear trapezoidal method. To assess the performance of models, ME, MAE, and RMSE were calculated using the observed data and predicted values from models. The formulas were defined as follows:

10 $$ME=\frac{1}{n}\sum_{i=1}^{n}\left({p}_{i}-{o}_{i}\right),$$

11 $$\mathrm{MAE}=\frac{1}{n}\sum_{i=1}^{n}\left|{p}_{i}-{o}_{i}\right|,$$

12 $$\mathrm{RMSE}=\sqrt{\frac{1}{n}\sum_{i=1}^{n}{\left({p}_{i}-{o}_{i}\right)}^{2}},$$

where n is the number of predicted data, and *p*_i_ and *o*_i_ represent the predicted and measured values, respectively. Models with lower ME, MAE, and RMSE values perform better. Unless otherwise specified, data are shown as mean ± standard error (S.E.) for the indicated number of animals. The pulse rate-dependency of AUE was evaluated by Dunnett’s test. The dose-dependency of the dose-normalized AUC was evaluated using the t-test. Statistical analysis was performed using JMP 14.3.0 software. A probability value of p < 0.05 indicates statistical significance.

## Supplementary information

Supplementary Information.
