# Supplementary material for: Pharmacokinetic–pharmacodynamic modelling of the hypoglycaemic effect of pulsatile administration of human insulin in rats
Source: Sci Rep. 2020 Nov 2;10:18876. doi: 10.1038/s41598-020-76007-3 (PMC7608663; doi:10.1038/s41598-020-76007-3)
Supplement: Supplementary file 1 — Supplementary Information. [file 41598_2020_76007_MOESM1_ESM.pdf]

Supplementary Material

Pharmacokinetic-pharmacodynamic modelling of the hypoglycaemic effect of pulsatile administration of human insulin in rats

Makoto Miyazaki, Mariko Hayata, Noriaki Samukawa, Kazunori Iwanaga, and Junya Nagai

Department of Pharmaceutics, Osaka University of Pharmaceutical Sciences, Osaka, Japan

Corresponding author:
Makoto Miyazaki Ph.D.
Present affiliation: Education and Research Center for Pharmaceutical Sciences, Osaka University of Pharmaceutical Sciences
4-21-1 Takatsuki, Osaka 569-1094, Japan
Tel.: +81-72-690-1299
Fax.: +81-72-690-1027
Email: miyazaki@gly.oups.ac.jp

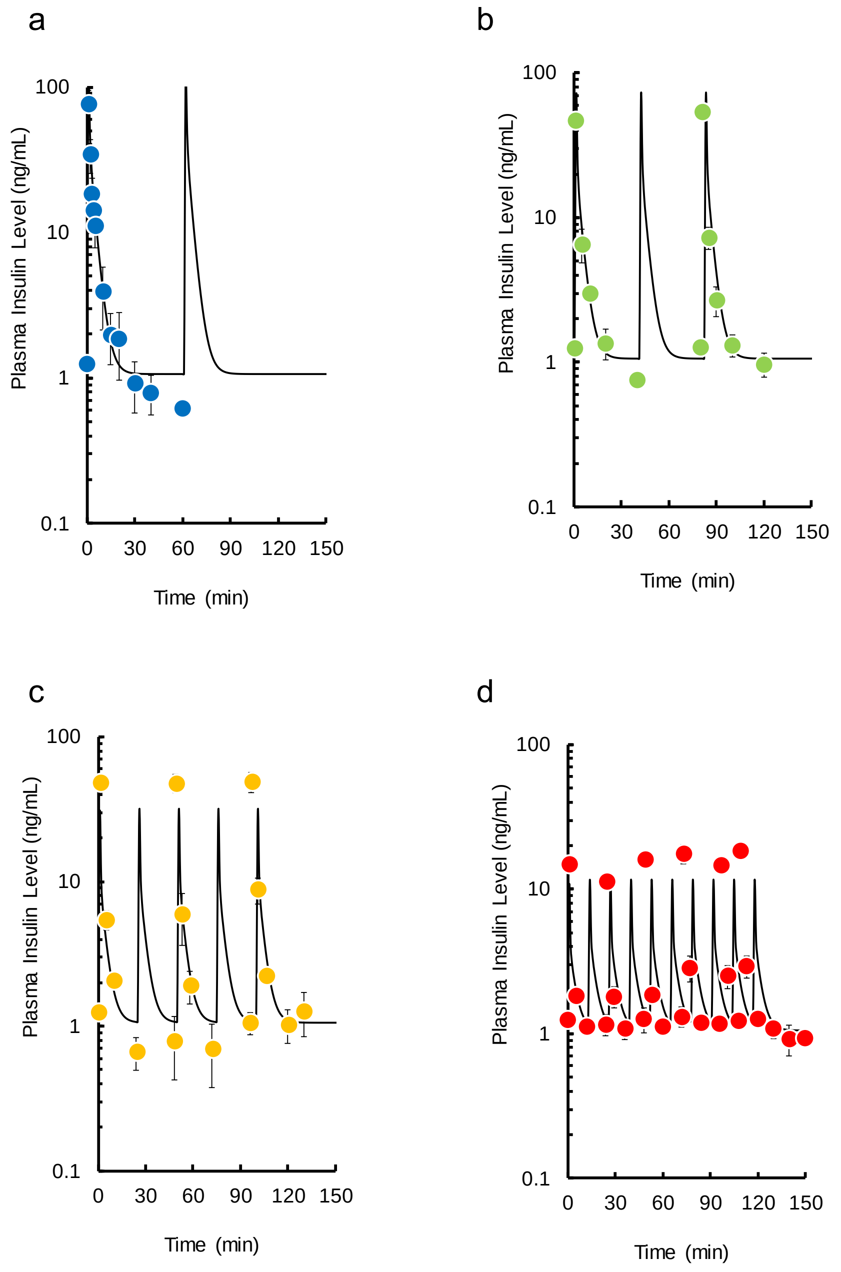

Supplementary Figure S1. a: The time courses of plasma INS concentration during and after intravenous pulsatile administration of INS (0.5 IU/kg/2 h) in rats as a: 2 pulses, b: 3 pulses, c: 5 pulses, d: 10 pulses. The points represent the mean ± S.E., n=3–5. The solid lines represent the model-estimated curves from INS concentrations for the single bolus administration data.

**Parameters**	**Estimated values**
Pharmacokinetic parameters	
k_{12} (min^{-1})	0.843 ± 0.25
k_{21} (min^{-1})	0.419 ± 0.073
k_{10} (min^{-1})	0.000314 ± 0.0079
Km (ng/kg)	1538 ± 624
Vmax (ng/kg/min)	3243 ± 649
Vc (mL/kg)	29.3 ± 6.6
Pharmacodynamic parameters of the IR model	
Kd (nmol/L)	2.062 FIX
SC_{50IR} (%)	7.60 ± 1.8
S_{maxIR}	3.01 ± 0.31
γ_{IR}	2.25 ± 1.8
k_{GoutIR} (min^{-1})	0.0208 ± 0.0018
Pharmacodynamic parameters of the GT model	
α	0.153 ± 0.56
T_{AB} (min)	3 FIX
T_{BA} (min)	20 FIX
SC_{50GT}	100.22 ± 354
S_{maxGT}	2.58 ± 1.3
γ_{GT}	0.736 ± 0.11
k_{GoutGT} (min^{-1})	0.141 ± 0.021

Supplementary Table S1. Pharmacokinetic and pharmacodynamic parameter estimates of the IR and GT models. k_{12}, k_{21}, and k_{10}, first-order rate constants for INS; K_m and V_{max}, Michaelis–Menten kinetic constants; V_c, volume of distribution for the central (plasma) INS compartment; K_d, binding affinity constant of INS; SC_{50IR} and SC_{50GT}, stimulation producing 50% of S_{maxIR} and S_{maxGT}, respectively; S_{maxIR} and S_{maxGT}, maximum effect of stimulation from IR model and GT model, respectively; γ_{IR} and γ_{GT}, Hill's constants of IR model and GT model, respectively; k_{GoutIR} and k_{GoutGT}, first-order rate constants of glucose elimination of IR model and GT model, respectively; α, proportional constant, T_{AB}, time required for GLUT4 to move to the cell surface; T_{BA}, time required for GLUT4 to be internalized in the cell. Data represents the computer-fitted value ± S.D.

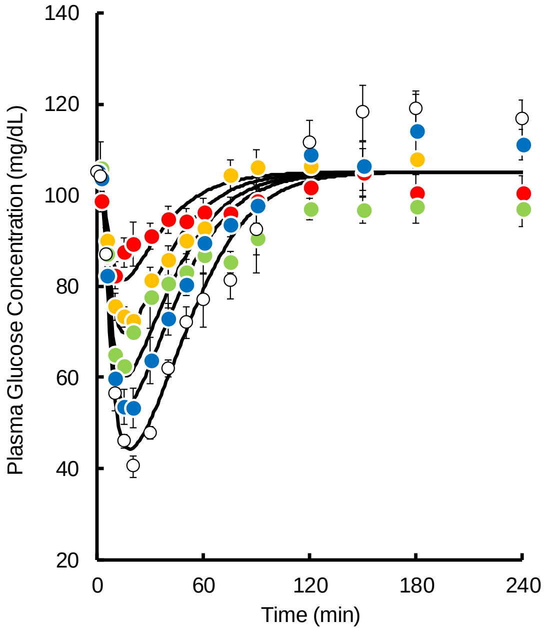

Supplementary Figure S2. The time courses of plasma glucose concentrations after a single intravenous bolus administration of INS in rats. The doses are 0.05, 0.1, 0.17, 0.25, and 0.5 IU/kg for red, orange, green, blue, and white, respectively. The points represent the mean ± S.E., n=3–5. The solid lines are the theoretical curves fitted to the IR-GT model.

	AIC	SS
Typical model	264.2744	30.4776
IR model	263.4786	30.1559
GT model	266.4003	30.5288
IR-GT model	238.0370	22.3178

Supplementary Table S2. Akaike Information Criterion (AIC) and sum of squares (SS) values at the time for fitting for the typical indirect response model (Fig. S3), IR model (Fig. 3b), GT model (Fig 3c), and IR-GT model (Fig. S2).

Parameters	***Estimated values***
Pharmacokinetic parameters	
k_{12} (min^{-1})	0.843 FIX
k_{21} (min^{-1})	0.419 FIX
k_{10} (min^{-1})	0.000314 FIX
Km (ng/kg)	1538 FIX
Vmax (ng/kg/min)	3243 FIX
Vc (mL/kg)	29.3 FIX
Pharmacodynamic parameters	
Kd (nmol/L)	2.062 FIX
α	1 FIX
T_{AB} (min)	3 FIX
T_{BA} (min)	20 FIX
SC_{50IRGT}	1276.56 ± 580.6
$S_{maxIRGT}$	4.59 ± 1.8
γ_{IRGT}	0.990 ± 0.020
$k_{GoutIRGT}$ (min^{-1})	0.236 ± 0.036

Supplementary Table S3. Pharmacokinetic and pharmacodynamic parameter estimates of IR-GT model. k_{12}, k_{21}, and k_{10}, first-order rate constants for INS; K_m and V_{max}, Michaelis–Menten kinetic constants; V_c, volume of distribution for the central (plasma) INS compartment; K_d, binding affinity constant of INS; SC_{50IRGT}, stimulation producing 50% of $S_{maxIRGT}$; $S_{maxIRGT}$, maximum effect of stimulation from IR-GT model; γ_{IRGT}, Hill's constant of IR-GT; $k_{GoutIRGT}$, first-order rate constants of glucose elimination of IR-GT; α, proportional constant, T_{AB}, time required for GLUT4 to move to the cell surface; T_{BA}, time required for GLUT4 to be internalized in the cell. Data represents the computer-fitted value ± S.D.

Model	Administration	ME	MAE	RMSE
IR model	10 pulses	-9.63<br>(-11.5, -7.80)	9.63<br>(7.80, 11.5)	11.1<br>(9.14, 13.1)
	Bolus	-2.95<br>(-7.93, 2.03)	7.61<br>(4.67, 10.5)	9.17<br>(6.23, 11.7)
GT model	10 pulses	8.88<br>(5.13, 12.6)	13.2<br>(11.3, 15.2)	14.5<br>(12.7, 16.3)
	Bolus	-6.91<br>(-10.8, -3.05)	8.29<br>(5.46, 11.1)	9.64<br>(7.02, 11.9)
IR-GT model	10 pulses	0.362<br>(-2.14, 2.87)	5.60<br>(3.90, 7.31)	7.64<br>(5.44, 9.49)
	Bolus	-2.25<br>(-6.03, 1.53)	5.34<br>(2.78, 7.90)	6.96<br>(4.24, 9.18)

Supplementary Table S4. Comparison of the prediction indicators of the three models for plasma glucose concentration. Numbers in parentheses indicate 95% confidence intervals. ME, mean error; MAE, mean absolute error; RMSE, root mean square error.

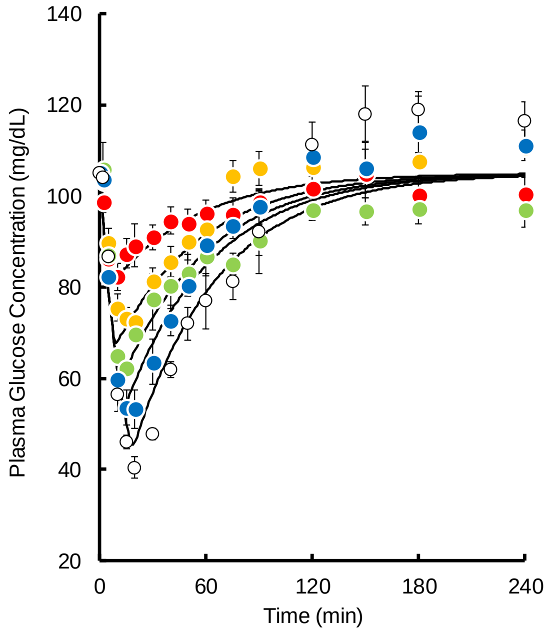

Supplementary Figure S3. The time courses of plasma glucose concentrations after a single intravenous bolus administration of INS in rats. The doses are 0.05, 0.1, 0.17, 0.25, and 0.5 IU/kg for red, yellow, green, blue, and white, respectively. The points represent the mean ± S.E., n=3–5. The solid lines are the theoretical curves fitted to the typical indirect response model.

Parameters	***Estimated values***
Pharmacokinetic parameters	
k_{12} (min^{-1})	0.843 FIX
k_{21} (min^{-1})	0.419 FIX
k_{10} (min^{-1})	0.000314 FIX
Km (ng/kg)	1538 FIX
Vmax (ng/kg/min)	3243 FIX
Vc (mL/kg)	29.3 FIX
Pharmacodynamic parameters	
SC_{50ty} (ng/mL)	2.18 ± 0.224
S_{maxty}	2.96 ± 0.263
γ_{ty}	12.8 ± 31.94
k_{Goutty} (min^{-1})	0.0203 ± 0.0187

Supplementary Table S5. Pharmacokinetic and pharmacodynamic parameter estimates of the typical indirect response model. k_{12}, k_{21}, and k_{10}, first-order rate constants for INS; K_m and V_{max}, Michaelis–Menten kinetic constants; V_c, the volume of distribution for the central (plasma) INS compartment; SC_{50IRty}, plasma INS concentration producing 50% of S_{maxty}, the maximum effect of stimulation; γ_{ty}, Hill's constants; k_{Goutty}, first-order rate constants of glucose elimination. Data represents the computer-fitted value ± S.D.
